# Impaired cardiac modulation in patients with functional seizures: Results from a face intensity judgment task

**DOI:** 10.1111/epi.17761

**Published:** 2023-09-12

**Authors:** Akihiro Koreki, Sarah Garfinkel, Hugo Critchley, Sarah Cope, Niruj Agrawal, Mark Edwards, Mahinda Yogarajah

**Affiliations:** ^1^ Neurosciences Research Centre St George's University of London London UK; ^2^ Department of Psychiatry National Hospital Organization Shimofusa Psychiatric Medical Center Chiba Japan; ^3^ Institute of Cognitive Neuroscience UCL London UK; ^4^ Brighton and Sussex Medical School Sussex University Sussex UK; ^5^ Atkinson Morley Regional Neuroscience Centre St George's Hospital London UK; ^6^ Department of Clinical & Experimental Epilepsy UCL Queen Square Institute of Neurology London UK; ^7^ Chalfont Centre for Epilepsy London UK; ^8^ NIHR University College London Hospitals Biomedical Research Centre London UK

**Keywords:** agency, dissociation, functional seizures, heartbeat, interoception

## Abstract

**Objective:**

Although interoceptive abnormality in patients with functional seizure (FSs) has been demonstrated using explicit tasks, implicit measurements of interoception such as the effect of interoception on perceptual brain processes have not been investigated. It has been shown that perception is normally modulated by interoceptive signals related to the different phases (systole vs diastole) of the cardiac cycle (cardiac modulation effect). Given our previous findings using explicit measures of interoception, we hypothesized that cardiac modulation would be impaired in FSs.

**Methods:**

Thirty‐two patients with FSs and 30 age‐ and sex‐matched non‐clinical individuals conducted a face intensity judgment task, in which their intensity rating when fearful or neutral faces was presented was compared between systolic and diastolic phases. They also conducted the heartbeat discrimination task as a measure of their capacity to integrate both interoceptive and exteroceptive information.

**Results:**

Patients with FSs had impaired cardiac modulation of the perception of neutral faces (corrected *p* = .044). Individual differences in the heartbeat discrimination task predicted the degree to which cardiac modulation occurred across the whole group (*p* = .028). This cardiac modulation effect was significantly associated with seizure severity (*p* = .021). Regardless of cardiac phase, patients rated fearful facial expressions as less intense compared to control participants (*p* = .006).

**Significance:**

These findings highlight impaired implicit cardiac modulation effects in patients with FSs. This reflects interoceptive dysfunction in patients with FSs, and an inability of the brain to integrate interoceptive signaling with perceptual processing. This may have implications for our understanding of the pathophysiology in FSs and inform novel diagnostic approaches.

**Key points**

- The intensity rating of faces presented at systole/diastole is an implicit interoceptive measure.
- In the task, impaired cardiac modulation was found in patients with functional seizures (FSs).
- The heartbeat discrimination task predicted the degree to which cardiac modulation occurred.
- This reflects interoceptive dysfunction in patients with FSs.
- It suggests an inability of the brain to normally modulate the effects of interoception on other brain processes.

## 1 INTRODUCTION

Interoception refers collectively to the body‐to‐brain processing of signals originating from the internal body and visceral organs.^1^ We have previously demonstrated that cardiac interoceptive trait abnormalities, as indexed by tests of heart beat perception or an individual's ability to explicitly (consciously) and accurately perceive their heart beats, are present in patients with functional seizures (FSs).^2^ We have also demonstrated dynamic interoceptive abnormalities in patients with FSs.^3^ Other groups have also demonstrated interoceptive abnormalities in FSs to varying degrees.^4^, ^5^ However, interpretation of deficits in performance accuracy on the heart beat counting task is constrained by its sensitivity to non‐interoceptive factors. For example, expectation and estimation are likely to contribute a subjective element to both the performance and the effects of interoceptive representation on emotional behavior.^6^, ^7^ Furthermore, these explicit tasks cannot assess direct, often pre‐conscious, effects of interoception on other cognitive or perceptual functions.

Experimental paradigms involving cardiac timing—in which the implicit effects of stimuli presented on systole are compared to stimuli presented on diastole—represent one means of addressing these limitations, by capitalizing on natural fluctuations in interoceptive signaling related to different phases of the heartbeat. These paradigms test the effect of presentation of a stimulus on systole vs diastole on perception or action. The detection of and responses to, visual, auditory somatosensory, and startling or painful stimuli,^8^ are attenuated at systole compared to diastole. Current models postulate that the ejection of blood from cardiac ventricles (systole) evokes baroreceptor firing which, when conveyed neurally to the brain, suppresses cortical excitability and attenuates the sensory processing of external stimuli.^9^ However, although processing of non‐affective stimuli is often improved at diastole, when the neural noise is reduced,^9^ the processing of some emotional stimuli, notably those that signal threat or fear, can be enhanced at systole, which is likely to be an adaptive, evolutionarily conserved response to “fight or flight” like behavior where the heart rate will typically rise.^10^, ^11^, ^12^ Relatedly, motor action may also be enhanced at systole compared to diastole, as indicated by inhibitory motor control task performance, muscle activity, and cortical motor excitability.^9^ Furthermore, the sense of agency is enhanced at systole.^2^, ^13^

The modulation of brain processes by fluctuating interoceptive signals from the cardiac cycle can be understood within the concept of dynamic gain modulation, or dynamic precision weighting in the parlance of predictive processing models of the brain.^9^ Gain modulation can boost processing of salient (important) information while attenuating processing of non‐salient (distracting) stimuli.^9^, ^14^ Here the brain putatively accommodates the continuously oscillating cardiac signal by optimizing perception and action through a precision‐weighting (or gain modulation) mechanism between interoception and exteroception, by changing the heart rate. Skora and colleagues draw upon earlier models^15^ to propose that when a situation demands high perceptual sensitivity (e.g., accurate visual perception), the relevant exteroceptive sensory channel is assigned high precision, whereas the precision of the cardiac interoceptive channel is reduced by a reduction in heart rate in order to attenuate the impact of each systole on concurrent processing.^9^ As a result the exteroceptive, as opposed to the interoceptive, channel dominates the resulting inference. Conversely when action is more important than perception, exteroceptive channels no longer need to be assigned higher precision relative to cardiac information, and heart rate climbs, facilitating motor action. This model proposes that perception, action, and the flexible modulation of one in relation to the other, which is essential to adaption to and survival in a changing environment, is underpinned by dynamic cardiac modulation effects. The exploitation of these cardiac modulation effects lends itself to implicit methods of interoceptive testing.^16^, ^17^ Disruption of these cardiac modulation effects may reflect impaired dynamic gain modulation (or dynamic precision weighting) by the brain of interoceptive and exteroceptive channels. Disturbance of interoception and cardiac modulation of perception is reported in patients with distinct psychiatric disorders, including depression, anxiety, borderline personality disorder, and, notably, schizophrenia.^11^ However, cardiac modulation effects on exteroceptive processing have not been explored previously in patients with FSs. We previously reported reduced interoceptive accuracy in patients with FSs using explicit cardiac interoceptive testing, including the heartbeat discrimination task, which requires integration of exteroceptive and interoceptive information.^2^ We, therefore, hypothesized that this finding may also be reflected in disrupted cardiac modulation of the emotional face intensity task.^11^, ^12^

## 2 METHOD

### 2.1 Participants

Thirty‐three patients with FSs and 32 age‐ and sex‐matched non‐clinical individuals (“healthy controls”; HCs) were recruited at St George's University/Hospital, which is a tertiary center for clinical neurosciences. The diagnosis of FSs was made according to International League Against Epilepsy (ILAE) diagnostic criteria by at least two clinicians experienced in the diagnosis of epilepsy. Patients with a dual diagnosis of FSs and epilepsy were excluded from this study to reduce the heterogeneity. Anxiety and depression were assessed using the State–Trait Anxiety Inventory (STAI)‐State,^18^ and the Beck Depression Inventory (BDI).^19^ All patients had also undergone a formal psychiatric assessment interview. The research governance sponsoring committee of Fulham, London Health Research Authority approved the study protocol (IRAS 231863, REC 18/LO/0328). The study was conducted in accordance with the ethical guidelines set forth by the Declaration of Helsinki. All participants gave written informed consent for the study. After excluding three participants whose data was compromised by equipment failure, the data of 32 patients with FSs and 30 HCs were analyzed.

### 2.2 Face intensity judgment task

**FIGURE 1:**
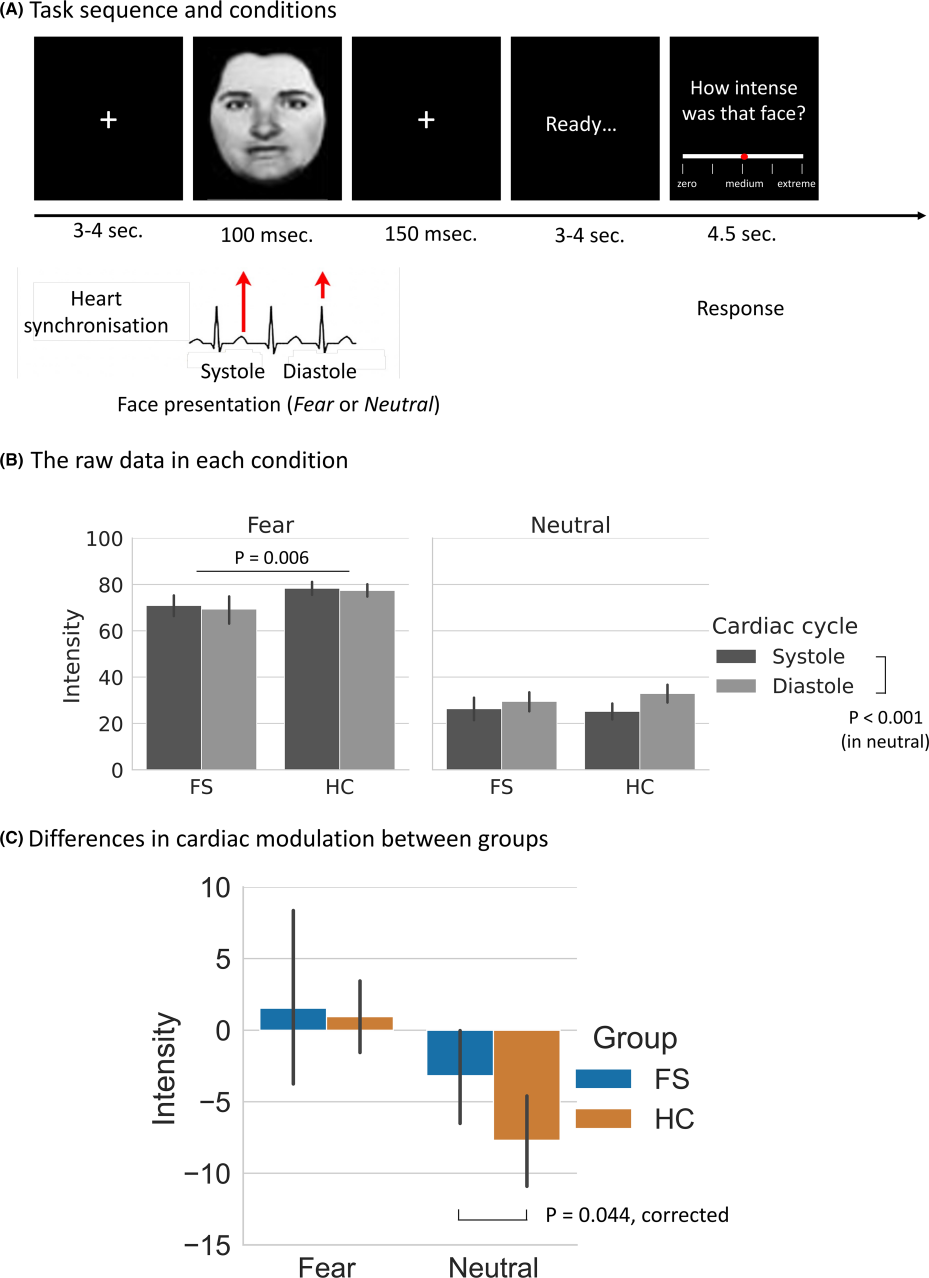
Trial sequence and cardiac modulation effects. (A) The task has two conditions (fear and neutral face) presented at distinct phases of the cardiac cycle: ventricular systole (when arterial baroreceptors fire) and diastole (when baroreceptors are quiescent). (B) Patients with functional seizures (FSs) rated fear faces significantly less intense than the non‐clinical comparison group (health controls [HCs]) (*p* = .006). Intensity ratings to neutral faces at systole compared to diastole, was significantly reduced in both groups (*p* < .001). Intensity ratings of fearful faces at systole was higher than at diastole in both groups but subthreshold for significance. (C) Intensity ratings of neutral faces were significantly reduced at systole compared to diastole in the HC but not in the FS group (corrected *p* = .044) even after controlling age, sex, heart rate, anxiety, and depression. Error bars are bootstrap 95% confidence interval).

This task is an implicit method of interoceptive testing and provided the primary measure in this study. Participants were asked to rate the emotional intensity of each presented face using a visual analog scale (VAS). During the experiment, the participant wore an oximeter (Nonin Xpod 3012LP) on their left index finger, from which pulse times were recorded to identify relevant periods in the cardiac cycle.^11^ Following published methods,^12^ facial images depicting two different emotional expressions (fearful and neutral) were presented time‐locked either to ventricular systole or diastole (Figure 1A). Face stimuli were taken from the Karolinska Directed Emotional Faces^20^: and the Ekman set.^21^ Presentation of the facial stimuli was brief (100 ms) to ensure that they were presented over target phases of the cardiac cycle. After each stimulus, the participant was asked to rate how “intense” they thought the face was. These emotional intensity judgments were logged by the participant using the VAS.^10^ The VAS ran from 0 to 100, and a marker started at the middle of the line. Each participant conducted 80 trials (40 fear and 40 neutral faces), randomized and balanced such that half of each emotion group was to coincide with systole and the other half with diastole. Checks were made offline as to whether stimuli were adequately presented during the target cardiac phases. Only trials with adequate presentation timing were analyzed. Processing of the task compares the difference of participants’ intensity rating between systolic and diastolic, and the difference is reflected in the cardiac modulation effect, where neutral stimuli would be especially diminished at systole, as explained above.

### 2.3 Heartbeat discrimination task (HDT)

This task is an explicit method of interoceptive testing in contrast to the face intensity judgment task described above. During performance of the HDT, the participant's pulse was monitored using the oximeter connected to a laptop computer that generated auditory tones based on their pulse. On each trial, the participant was instructed to listen to 10 tones and then report whether the tones occurred synchronously or asynchronously with their own heart beats.^11^ Interoceptive accuracy for HDT was calculated over 20 trials as a ratio of correct to incorrect synchronicity judgments.

### 2.4 Statistics

Baseline characteristics were compared between FS and HC groups using a two‐tailed *t* test, Mann–Whitney *U* test, or a chi‐square test depending on the data type. Reported intensity was analyzed separately in fear and neutral conditions. A two‐way repeated‐measures analysis of variance was used to look for an interaction between group and cardiac cycle phase, as well as the effect of each on fear or neutral face intensity ratings. Intensity in fear and neutral conditions was set as a dependent variable.

As our main analysis in the present study, cardiac modulation effect was compared between groups. To adjust for background differences between groups, an analysis of covariance was conducted with the cardiac modulation effect as the dependent variable, and age, sex, heart rate, anxiety, and depression as independent variables. Here, following published data, we defined the cardiac modulation effect in neutral = intensity in diastole – intensity in systole, and the cardiac modulation effect in fear = intensity in systole – intensity in diastole. Because both scores of anxiety and depression were not‐normally distributed, the presence or absence of clinically significant anxiety (defined by STAI‐State ≥40), and presence or absence of depression (defined by BDI ≥14) were included within the model.^18^, ^19^ Here, because two separate models were used (fear and neutral), a false discovery rate (FDR) correction was employed for multiple comparisons.

Furthermore, we conducted a correlation analysis between *total* cardiac effect and HDT. Here, the total cardiac effect is the sum of the cardiac modulation effects described above. One patient was excluded from this correlation analysis due to missing HDT data. A multi‐level correlation analysis was conducted with group as a random effect. We also carried out correlational analyses between the cardiac modulation effect and seizure severity in FSs. Seizure severity was defined by multiplication of frequency and duration. Two patients were excluded from this analysis due to lack of clinical data. Statistical analyses were carried out using R (4.1.3). A *p*‐value of <.05 was considered statistically significant.

## 3 RESULTS

### 3.1 Characteristics of participants

Both groups were well matched for age (29.5 ± 10.0 in FS vs 31.9 ± 10.5 in HC, *p* = .362) and sex (male and female: 2 and 30 in FS vs 3 and 27 in HC, *p* = .666). Heart rate at rest was faster in FS than in HC (mean ± SD: 73.2 ± 10.8 bpm in FS vs 64.0 ± 9.6 bpm in HC, *p* < .001). Heart rate during task was also faster in FS than in HC (74.0 ± 10.8 bpm in FS vs 68.6 ± 7.7 bpm in HC, *p* = .027). Anxiety (median 44 [interquartile range (IQR): 22.5] in FS vs 28 [8.8] in HC, *p* < .001) and depression (median 20.5 [20.5] in FS vs 2.5 [5.8] in HC, *p* < .001) were more severe in FS than HC. Patients were clinically characterized in the following ways: mean seizure onset age was 25.0 ± 10.8 years old; the mean duration of illness was 4.7 ± 4.2 years; the frequency of seizures was a median 1.0 [6.8] times/week; the duration of seizures was a mean 7.0 [22.0] min; the level of diagnostic certainty^22^ was that 10 patients had a clinically established, 15 a documented, and 2 a probable diagnosis; and finally in respect of other functional co‐morbidities, 1 patient had an associated functional sensory disturbance and 1 patient had associated functional weakness.

### 3.2 Performance of the face intensity judgment task

In the task, the total number of trials with adequate presentation timing were not different between groups (51.4 ± 22.3 in FS vs 52.7 ± 21.6 in HC, *p* = .819). Our statistical model for the condition of neutral face revealed no significant differences between the groups (*F*_1,58_ = 0.165, *p* = .686), but there was a significantly lower intensity rating at systole compared to diastole (*F*_1,58_ = 23.200, *p* < .001), and a significant interaction between groups and cardiac cycle (*F*_1,58_ = 4.094, *p* = .048) (Figure 1B). That is, the observed reduction in intensity rating was significantly attenuated in FS compared to HC participants when viewing neutral faces at systole compared to diastole. Our analysis of the fearful face condition revealed a significant reduction in the rating of fear faces in FS compared to HC participants (*F*_1,58_ = 7.998, *p* = .006), but no significant difference between cardiac cycle (*F*_1,58_ = 0.503, *p* = .481), and no significant interaction between group and cardiac cycle (*F*_1,58_ = 0.029, *p* = .866).

### 3.3 Comparison of cardiac modulation effect between groups

Our model for the cardiac modulation effect for neutral faces showed a significant group difference (*F*_1,55_ = 5.593, *p* = .022), even after controlling for age, sex, heart rate, anxiety, and depression. This result remained significant after FDR correction (corrected *p* = .044). There was no significant group difference in the cardiac modulation effect for fearful face stimuli (Figure 1C).

### 3.4 Correlation between HDT accuracy and total cardiac modulation effect

**FIGURE 2:**
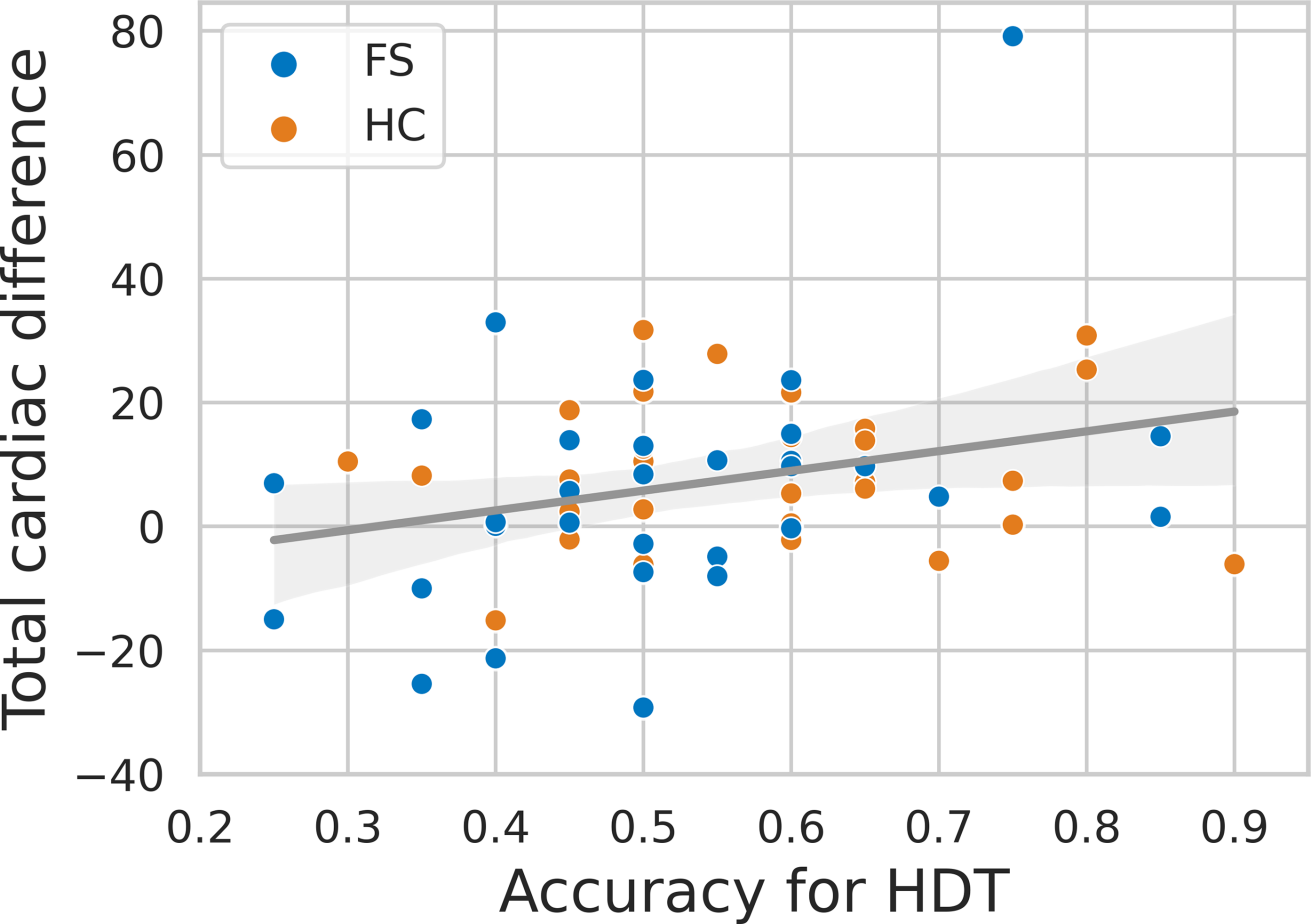
A correlation analysis between total cardiac effect and interoceptive accuracy. Performance of a heartbeat discrimination task (HDT accuracy) predicted total (i.e., for both fearful and neutral faces) cardiac modulation effects (*r* = .28, *p* = .028).

Although performance accuracy in the HDT was poorer on average in patients with FS (proportion correct = 0.51 ± 0.15) relative to HC participants (proportion correct = 0.57 ± 0.14), this difference did not meet threshold significance (*p* = .117). Nevertheless, one‐sample *t* tests showed that patients performed no better than chance (50%), in contrast to the HC group who did perform significantly better than chance (*p* = .008). Our multi‐level correlation analysis revealed that HDT accuracy predicted total (fear and neutral) cardiac modulation effect (*r* = .28, *p* = .028) (Figure 2).

### 3.5 Association between clinical information and total cardiac modulation effect

**FIGURE 3:**
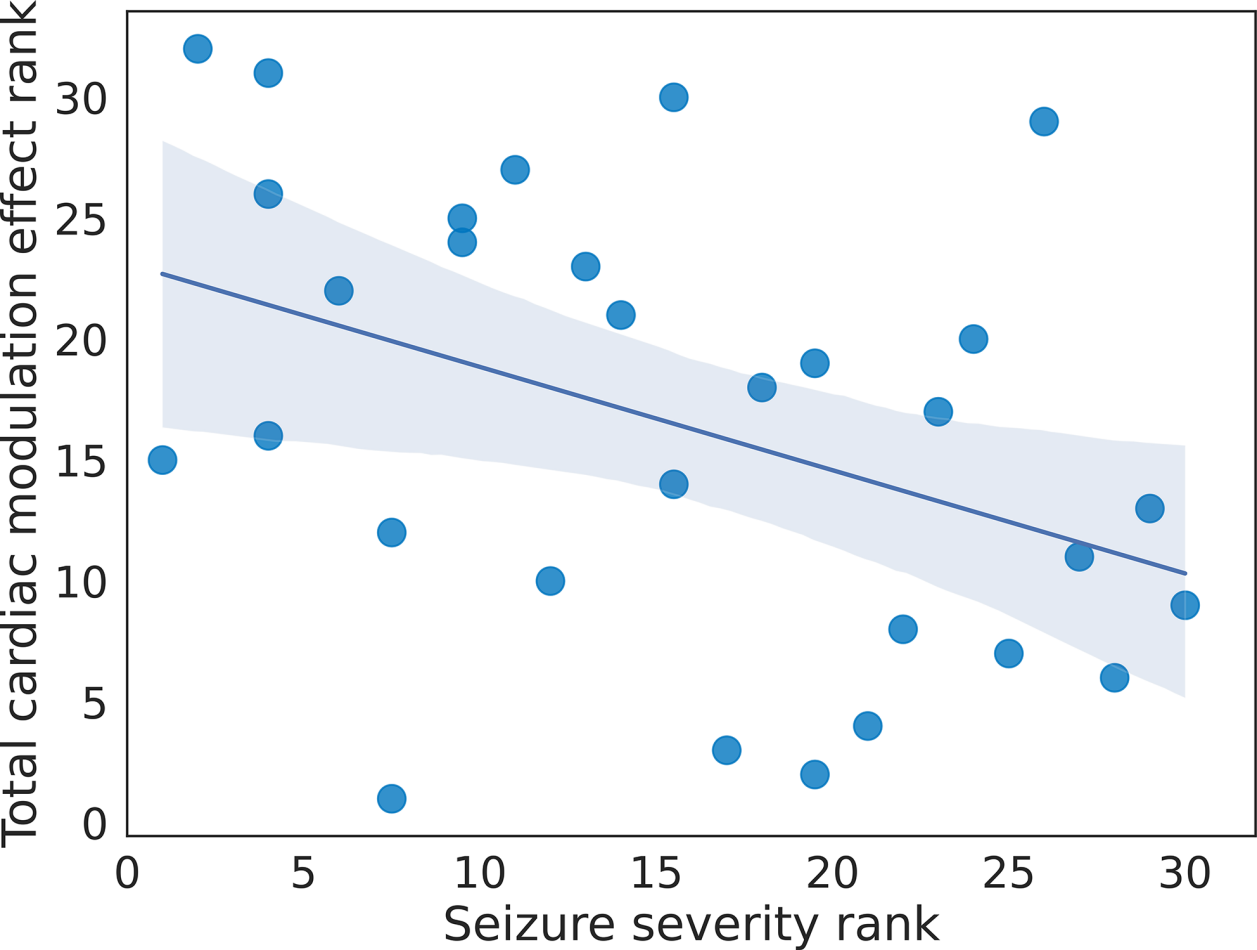
A correlation analysis between total cardiac effect and seizure severity. Seizure severity rank was plotted against total cardiac modulation effect rank. Seizure severity was defined by multiplication of frequency and duration. Both were significantly correlated (rho = −.410, *p* = .021). That is, the greater levels of impaired cardiac modulation were associated with more frequent and longer‐lasting functional seizures.

Total cardiac modulation effect was significantly associated with severity of FSs defined by multiplication of frequency and duration (rho = −.410, *p* = .021) (Figure 3), with a trend‐level correlation with frequency (rho = −.336, *p* = .064) and no correlation with duration (*p* = .774). That is, the greater levels of impaired cardiac modulation were associated with more frequent and longer lasting FSs.

## 4 DISCUSSION

To the best of our knowledge, this is the first study to examine the automatic, arguably preconscious, impact of interoceptive signals on the perceptual processing of socially salient information in patients with FSs. In testing how cardiac afferent signals influence the perception on affective intensity ratings of neutral and fearful facial expressions, in agreement with the hypothesis, we found patients with FSs show reduced cardiac modulation of how neutral faces are perceived. We also found the supporting evidence that individual differences in explicit heartbeat perception (quantified by using a task requiring integration of interoceptive and exteroceptive information) predict the degree to which heartbeat signals at systole influence facial processing. Of interest, the cardiac modulation effect was associated significantly with severity of FSs. This suggests that this effect may be related to the underlying pathophysiology of FSs. We also replicated the differential effect of cardiac phase on fearful vs neutral faces, showing the reduction in facial intensity ratings at systole compared to diastole when viewing neutral faces in HCs.^11^, ^12^ This finding reinforces the general concept that cardiac afferent signaling can interfere with perceptual processing (here of faces), which typically improves at diastole due to reduced interoceptive noise from baroreceptor firing.^9^ Furthermore, as an additional finding, patients with FSs rate fearful facial expressions as less intense (regardless of cardiac phase) than non‐clinical individuals.

As hypothesized, patients with FSs demonstrated a significantly reduced cardiac modulation effect on the perceived intensity of neutral faces. A previous transdiagnostic study^11^ demonstrated attenuated cardiac modulation of neutral faces in depression, anxiety, bipolar disorder, borderline personality disorder, and schizoaffective disorder. Although it is difficult to compare our observations in FSs directly with these other conditions because of the different experimental settings, it is worth noting that our findings in the present study were independent of anxiety and depression. That is, our statistical model showed a significant group difference even after controlling for anxiety and depression, suggesting that the observed difference cannot be attributed solely to anxiety and depression, and therefore might appear more specific to the diagnosis of FSs, although further study is required to consider other possible confounding factors. In contrast to neutral faces, the affective rating of fearful faces showed a trend to an increase at systole compared to diastole. However, in contrast to earlier studies in HCs,^11^, ^12^ the difference did not reach statistical significance, possibly because of small numbers of participants. Although emotional face processing may be associated with interoceptive firing,^12^, ^23^ further research is needed to understand how the affective value of fearful faces and other threat cues relates to subjective judgments interacting with cardiac phase and efferent autonomic control.^9^, ^15^, ^24^

A significant correlation across both groups between total cardiac difference and HDT accuracy indicates that the degree of cardiac modulation when viewing faces is associated with an individual's ability to consciously integrate information across exteroceptive and interoceptive channels, as indexed by the HDT. In this cardiac interoceptive task, participants need to judge whether they experience (exteroceptive) auditory tones as coincident or delayed relative to their own heart beats (interoception). In one proposed model,^9^ the differential rating of faces viewed on systole vs diastole reflects the brain's active modulation of interoeptive and exteroceptive channels. On this basis it is, therefore, unsurprising that we see a correlation between HDT performance and the total cardiac modulation effect. Perception, action, and the flexible modulation of one in relation to the other, is adaptive, enhancing survival in a changing environment. The impaired cardiac modulation observed in FSs may therefore reflect a breakdown in this active regulatory mechanism, and potentially a relevant contributory factor to the seizures themselves. Indeed, in our previous study of FSs, we reported that deficits in interoceptive accuracy, specifically measured by the HDT, predict seizure frequency.^2^ The breakdown in cardic modulation effects reported here may thus arise as an inability to coordinate exteroceptive and interoceptive channels.

The impaired cardiac modulation observed in the current perception task may also have implications for action: Cardiac modulation, facilitating switching between perception and action, can be harnessed to optimize both perception and action.^9^ Correspondingly, the findings reported here support previously reported positive links between interoception and action (conversely, impaired interoception is linked to aberrant action). A number of studies report that inhibitory control, muscle activity, and sense voluntariness/agency are associated with facilitatory effects of systole under normal cardiac modulation.^2^, ^13^, ^25^, ^26^, ^27^, ^28^ Given these observations, compromised cardiac modulatory influence may foster the expression of uncontrolled or impaired movements with a reduced sense of agency, arguably a hallmark of certain functional movement disorders, notably FSs.

Of interest, independent of cardiac modulatory effects, we observed that fearful facial expressions were judged less intense by patients with FSs than by HCs. Core interoceptive deficits may also underlie this effect in FSs^2^ wherein emotional feelings states are, according to “peripheral theories of emotion,”^29^, ^30^ fundamentally based on internal physiological changes. Correspondingly, individuals with better interoceptive accuracy are sensitive to the emotions of others.^31^ Moreover, enhancement of interoceptive bodily states of fear may facilitate the recognition of the fearful faces.^32^ Altered emotional processing is reported across functional neurological disorders (including FSs) (for a review, see Ref. 33). In FSs, patients show deficits in the recognition of facial emotional expressions.^34^ An imaging study using an emotional face task showed a hyperactive limbic system in functional neurological disorder (for a review, see Ref. 33), including FSs.^35^ Interoception also plays a key role in emotional regulation.^36^ Poor emotional processing is associated with alexithymia,^37^ that is, the impaired ability to recognize and describe one's own emotional state, which itself may have a basis in attenuated interoception. In this context, evoked bodily states of hyperarousal may contribute to the “panic attack without panic” commonly associated with the expression of FSs.^38^

A key strength of the current study is that assessment of interoceptive effect on perception is implicit, harnessing the cardiac modulation of a face intensity judgment task. This approach overcomes the inherent limitations of explicit cardiac interoceptive behavioral testing, for which inference is constrained by potential confounding effects of prior beliefs and expectations. Furthermore, this task enables the assessment of dynamic changes in brain functioning based on interoceptive firing. Despite these advantages, several limitations to the current study remain. First, direct cardiac modulation effects for action cannot be assessed using the present task. Second, our study has a small sample size. Third, the current task cannot assess the cardiac effects on other emotional faces, such as disgust, angry, and happy. Fourth, we did not collect any history pertaining to previous traumatic events or stressors and cannot, therefore, explore their contribution to the observed effects. Nonetheless, we would argue that this study has focused on underlying proximal pathophysiological mechanisms underpinning functional seizures, regardless of more distal risk or vulnerability factors for the development of functional seizures. Finally, given the use of two separate models of the effect of group on cardiac modulation of either neutral or fearful faces, there is a risk of type I error. To deal with this limitation, a larger replication study is needed in the future.

## 5 CONCLUSION

Impaired cardiac modulation in FSs was found in the present study using the face intensity judgment task. We believe that it sheds light on the pathophysiology in FSs and may inform novel diagnostic and therapeutic approaches.

## AUTHOR CONTRIBUTIONS

S.G., H.C., M.E., and M.Y. designed the study. A.K. and M.Y. conducted data collection and analyzed the data. S.C., N.A., and M.E. developed the research environment and helped with data collection. S.G., H.C., and M.E. provided technical and conceptual suggestions. A.K. and M.Y. wrote the first draft, and all co‐authors have approved the final manuscript.

## CONFLICT OF INTEREST STATEMENT

None of the authors has any conflict of interest to disclose.
